# Expression of Concern: BlockTicket: A framework for electronic tickets based on smart contract

**DOI:** 10.1371/journal.pone.0350246

**Published:** 2026-05-27

**Authors:** The PLOS One Editors

The *PLOS One* Editors issue this Expression of Concern because this article [1] was identified as one of a series of submissions for which we have concerns about the peer review process. Readers are advised to interpret the article [1] with caution.
