# Supplementary material for: BlockTicket: A framework for electronic tickets based on smart contract
Source: PLoS One. 2023 Apr 12;18(4):e0284166. doi: 10.1371/journal.pone.0284166 (PMC10096233; doi:10.1371/journal.pone.0284166)
Supplement: Attachment — Submitted filename: Response to Reviewers.docx [file pone.0284166.s001.docx]

**BlockTicket: A Framework for Electronic Tickets Based on Smart Contract**

Amjad Aldweesh

Dear Editor,

We would like to thank the reviewers for their diligent and thorough reading of this paper, as well as their intelligent comments and helpful suggestions, which helped to improve its quality. We are pleased to notify you that we have updated the manuscript in response to the reviewers' recommendations. We believe that the updated manuscript is in much better shape after incorporating the feedback. We appreciate the reviewer's forthright comments: we recognized that some crucial paragraphs of the original article lacked clarity, with ambiguities that led to reader misunderstandings. We have now updated the manuscript's organization and included more paragraphs. More accurate mathematical formulas, we agree, clarify the description. The references have been updated in the new version. We have finally completed a thorough editing and formatting process. This update, we believe, improves the manuscript's readability. The reviewer's comments are addressed in detail below.

We hope that these revisions improve the paper such that the reviewers now deem it worthy of publication in your esteemed journal. For the reviewers' convenience, we have followed their comments by our response below. The uploaded copy of the original manuscript marked with all the changes made during the revision process. The new text is highlighted in "red colour".

Thank you

Amjad

**Reviewers**:

1. “This paper proposes an E-ticketing framework that utilizes blockchain technology. By separating users’ credential information from financial transactions, the blockchain-based electronic ticketing model reduces the risk of data leaks and enhances privacy as well as removing third party involvement. Further there are some major parts that are missing in order to accept the paper.”
2. “In this research, the authors proposed an E-ticketing framework that utilizes block chain technology. By separating users’ credential information from financial transactions, the block-chain-based electronic ticketing model reduces the risk of data leaks and enhances privacy as well as removing third party involvement. The work is solid and substantive, but there are some points in the purpose for improvement.”
3. “The author utilizes blockchain implementation for the E-ticketing framework. The proposed approach improves throughput, decreases redundant work, and increases consensus efficiency. Also, the analysis of experimental data demonstrates the framework's benefits, which include fast ticket holding times, high throughput, and flexible scalability. The overall quality of this paper is acceptable, but I have the following comments for improvement.”

**Reply**: Thank you for agreeing with us on the aim of this manuscript. We attentively studied your comments and did our best to answer them one by one, particularly in terms of improving the presentation. We hope that this revision has enhanced the work to “Plos One” standard.

**Comment**: The related work section is very weak, i recommend the author should include the tables for the related review from year 2021-22. I recommend the authors should do a critical analysis of the problem, available solution, drawbacks in current framework for which author have written manuscript. Also used the latest research articles form 2021 & 2022 for performing related work.

**Reply**: We appreciate the guidance. We have extended the related work and we also included the table. Please refer to the submitted revised version line [201-233].

**Comment:** The abstract needs to be rewritten more precise and concrete. Including, Introduction, Objective, Method, Results, Conclusion. Please improve the abstract, it should list all the contributions made very clearly.

**Reply:** Thank you for directing us in the right direction. We appreciate the reviewer's encouragement, we rewrote the abstract and included Introduction, Objective, Method, Results, Conclusion.

**Comment:** The manuscript lack in detailed description of methodology section and a flow diagram of the work. The methodology section is the core of the work, so it must be very well explained, there can be no doubts. The author must include separate methodology section and present a flow diagram of the work.

**Reply:** We thank the reviewer in supporting our intention, we added separate methodology section and present a flow diagram of the work. Please refer to the submitted revised version line [247-274].

**Comment:** Manuscript lacks in comparison of the results obtained with state of art methods or models.

**Reply:** Thank you so much for your comments that help us to improve our work significantly. We have updated the result to address comments.

**Comment:** The results section is very week to support the claim made by authors.

**Reply:** Thank you so much for your comments that help us to improve our work significantly. We have improved the results section. Please refer to line [443- 452].

**Comment:** The conclusion section needs to elaborate more by discussion the disadvantages of the developed framework & discussion on the results obtain. The author should also include the future work section.

**Reply:** Thank you for directing us in the right direction. We have extended the conclusion section to address the future work. Please refer to [525-end].

**Comment**: English required more improvement.

**Reply**: We appreciate the guidance. We thank the reviewer in supporting our intention. The paper is checked. We have revised the WHOLE manuscript carefully. We tried to improve the presentation and to avoid any grammar or syntax error. Please refer to the revised manuscript.

**Comment**: The quality of some figures needs to be enhanced. The author(s) must redraw them with high quality. Some text on figures is difficult to read.

**Reply**: We appreciate the guidance. We have updated Figures and we hope it now looks much more clear.
