# Supplementary material for: BlockTicket: A framework for electronic tickets based on smart contract
Source: PLoS One. 2023 Apr 12;18(4):e0284166. doi: 10.1371/journal.pone.0284166 (PMC10096233; doi:10.1371/journal.pone.0284166)
Supplement: Attachment — Submitted filename: ResponseR2.docx [file pone.0284166.s002.docx]

Dear Reviewers,

Thank you very much for your review and feedback on our paper. We appreciate your constructive feedback and suggestions for improvement. We will take them into account in revising our paper. Please refer to the following.

**Reviewer #3:** Kudos in the good work.

**Comment:**

Nevertheless, the authors can further explain more about the data (or dataset) used (such for verification and validation) to support this research. With the dataset used for evaluation being highlighted, this would substantiate all data underlying the findings.

**Rely:** Thank you for taking the time to review our paper. We are pleased to hear that you think the paper has improved since the last revision. We will make sure to address the minor points you have raised.

**Reviewer #4:**

The paper is improved since last revision. Some minor points still need to be addressed.

**Reply**: Thank you for taking the time to review our paper. We are glad to hear that you think the paper has improved since the last revision. We appreciate your constructive feedback, and we will make sure to address the minor points you have raised. We value your input, and we will do our best to make any necessary improvements in the next revision.

**Comment:** In table 1, use the name of the framework instead of ours.

**Rely:** Thank you for taking the time to review our paper, we have updated the mentioned table and including the name on that table. Please refer to the updated manuscript.

**Comment:** The visibility of Figures is low, Figures with better quality should be included.

**Rely:** We are sorry that the visibility of the figures in the paper is low. We understand the importance of clear and high-quality figures in presenting our results effectively. We will take your feedback into consideration and make sure to include figures with better quality in the updated manuscript.

**Comment:** Algorithms and figures should be referred in text close/near to Figures and Algorithms.

**Rely:** We apologize for not referring to the algorithms and figures in the text close to them. We will make sure to include references to the algorithms and figures in the text near their placement in the next revision. We appreciate your feedback, and we will do our best to improve the paper based on your suggestions. Please refer to the updated manuscript.

**Reviewer #5:**

Overall, the idea of manuscript is average, furthermore, the contribution is not adequate at the moment. The manuscript needs significant work.

**Reply**: Thank you for taking the time to review our manuscript. We appreciate your feedback, and we understand your concerns regarding the idea of the manuscript and its contribution. We are sorry to hear that you think the contribution of the manuscript is not adequate now. We understand the importance of clear and significant contributions in the field of research, and we will make sure to address this in the next revision. We appreciate your honesty and constructive criticism, and we will do our best to improve the manuscript based on your suggestions. We believe that with your feedback, we can make the necessary changes to ensure that the manuscript meets the standards of the field.

**Comment:** What are the limitations of the related works?

**Rely:** We would like to thank the reviewer for their valuable feedback, and we agree with those comments. We have added the limitations of the previous work and stated in the text with red highlighted colour.

**Comment:** Are there any limitations of this carried out study?

**Rely:** Thank you for taking the time to review our manuscript and for your valuable feedback. We appreciate your comments and suggestions, we added the limitations of the proposed frameworks. Kindly refer to the conclusion section.

**Comment:** How to select and optimize the user-defined parameters in the proposed model?

**Rely:** We thank the reviewer for their feedback that indeed improve the final presentation of the paper, and we would state that in smart contract and blockchain system, user defined parameter optimized by the logic or the smart contract during the testing we have evaluated those parameters and it shows that the wallet that we supposed being used by every entities does the optimization and doesn’t allow for invalid inputs.

**Comment:** Explain the evaluation metrics and justify why those evaluation metrics are used?

**Rely:** We appreciate your valuable comment, we have updated the manuscript to address this comment. Please refer to evaluation section

**Comment:** Some sentences are too long to follow, it is suggested that to break them down into short but meaningful ones to make the manuscript readable.

**Rely:** Thank you for taking the time to review our manuscript and for your helpful comments. We appreciate your suggestion to break down some of our longer sentences into shorter, more easily digestible ones. We agree that this will improve the readability of our manuscript and make it more accessible to a wider range of readers. We have proofread the manuscript and took into consideration your valuable comments.

**Comment:**

The title is pretty deceptive and does not address the problem completely.

**Rely:** We thank the reviewer for their comments, also, we agree with them. However, we are not sure about the ability to change the title regarding the journal policies.

**Comment:**

The related works section is very short and no benefits from it. I suggest increasing the number of studies and add a new discussion there to show the advantage. Following can be added: Use Anova test to record the significant difference between performance of the proposed and existing methods.

**Rely:** We agree with reviewer, and we have updated the related work section. Please refer to the updated manuscript.
