# Supplementary material for: BlockTicket: A framework for electronic tickets based on smart contract
Source: PLoS One. 2023 Apr 12;18(4):e0284166. doi: 10.1371/journal.pone.0284166 (PMC10096233; doi:10.1371/journal.pone.0284166)
Supplement: Attachment — Submitted filename: plosone revi.docx [file pone.0284166.s003.docx]

Dear Reviewer,

Thank you for your comment on the importance of a clear and concise presentation in research writing and figure quality. I completely agree with you that the introduction is a critical component of any research paper as it sets the stage for the entire study as well as the methodology and the quality of figures. We have addressed all your valuable comments, please refer to the updated manuscript.

**Comment (1):** Introduction: Your introduction should provide a clear and concise overview of the research problem, the significance of the problem, and the research questions or hypotheses that you are trying to address.

**Reply:** We would like to thank the reviewer for their valuable comment, we have improved this section kindly refer to updated manuscript line [44-52].

**Comment (2):** Methodology: Your methodology should describe the methods you used to collect and analyse your data. It should be clear and detailed enough to allow other researchers to replicate your study.

**Reply:** We appreciate the reviewer comments, we would like to inform you that the methodology section has been rewritten in an academic style to meet your valuable comment.

**Comment (3):** Figure 1 show be redrawn to be clearer.

**Reply:** We thank the reviewer for this comment which indeed improve the final presentation of the paper, the figure has been updated.

**Comment (4):** Some typos and grammar mistake that need to be addressed.

**Reply:** We have proofread the full paper and we believe it now looks better. Appreciate your comment.

Regards,

Author
